# Behind the Counter

**Published:** 1893-06

**Authors:** 


### BEHIND THE COUNTER.

### No. 6.

The floor walker within whose supervising limits my present duties lie is a good deal of a wag. Notwithstanding his readiness at all times to assist the novice, and smooth the way of the transgressor, if the offense is of a nature to be condoned, it sometimes happens that discipline fails to make any lasting impression upon the subject of it, and the final lesson to be applied, however reluctantly, is a dismissal. Among the more youthful assistants at the stationery counter was one of this class, who repeatedly drew attention to herself by her flippancy of manner and careless inattention, even when waiting upon customers. I had frequently observed the floor walker pause in his unceasing round, and even go out of his way to admonish her, but to little purpose, and 'ere long another had taken her place. I knew well enough that she had fallen under the ban of the very one whose warnings had passed unheeded ; whose duty, indeed, forced him to give " the nod," though his bald pate forbade any shaking of ambrosial curls. So, at a convenient moment, I alluded to the circumstance in an inquiring sort of a way, but the only reply was a comical stare and in great soberness this, from the author with whose works we form an early acquaintance:

> "There are some little women, and what do you think?
> They live upon nothing but victuals and drink.
> Though victuals and drink is the chief of their diet
> Yet these little women will never keep quiet."

I felt that I was answered.

The position of floor walker is not always an agreeable one. He is at once the director and regulator of affairs within his assigned limits, and the fitness and order of affairs depend largely upon his direction. It certainly requires a person of great aptitude and discrimination to fill the place to the satisfaction of both principal and subordinate. He can have no favorites—at least ostensibly—and familiarity with a subordinate is out of the question. Ever on the alert, he must be quick to see and swift to act whenever circumstances require his jurisdictional interference. He is at once overseer, monitor and arbitrator of affairs, from whose decision there is no appeal. He must sign, or countersign, all slips granting special privileges to either customers or employees, and accordingly as he carries himself will he be estimated. Even the cash girls—that little army of go-betweens—sum him up with discriminating acuteness, and their likes and dislikes are manifested in various ways. It is either "Old Snooks," or "Goodie Brown," a frown or a smile, whenever he gives one an order or interferes to settle a dispute.

As a rule, floor walkers command good pay, for only those who fill the position acceptably are retained. Some leave, never more to be of us, whilst others, promoted from the sales counter, find their way back there as by a natural selection. It is, after all, the "survival of the fittest," as Professor Darwin says of men and monkeys.

I have become quite reconciled to my new place, wherein I have acquired a commendable degree of efficiency; at least my superior, who is addressed as Miss Hopkins, awards me this credit. Perhaps it is because I had previously had a limited experience with the same line of goods.

I have had to undergo the ordeal of a novice, by being made the mark for an occasional harmless joke. It was only the other day that a healthy pattern of the Celtic race rushed up to me, in a great fluster, with "Schure, nouw, I was douwn to the far end o' the sthore, an' I lost me umbrella, an' the sthore walker said it was ye that had it." I

directed her to the office for lost packages, and as she turned to go, I caught a glimpse of our perambulating monitor, trying his best to look lamb-like and unconcerned, but his evident struggle betrayed him.

The afternoon of Thursdays is usually the maid-servants' weekly outing time, when they throng the dry goods stores, with their scanty means, and insist upon a very general inspection of the entire stock. If it were not amusing to observe their bungling efforts at gentility, the half day would be void of any enlivening feature.

I have made no allusion to Walter, my steadfast friend, for a considerable time, but it is not because we have not met after the old fashion, and spent many a profitable evening together, with some of the best authors for company. One of our late guests was Dr. Samuel Johnson, who entertained us with his Rasselas, whose opening leaves are said to embrace the very perfection of English composition; but I must confess I did not fully appreciate the descent from Happy Valley to the Sorrows of Werther, the love-lorn hero, who was weak enough to break his heart over another man's wife. Such a sentiment is only fit for idiots. There is no romance in it, and no common sense, and I told Walter plain enough that I didn't care if the story was a classic, and the very finest ever written, the sentiment was abominable, and that if he was silly enough to put himself in Werther's place, I wasn't disposed to play the part of another man's wife. At this Walter laughed outright, and without releasing my hand declared that it was the very last thing he could ever wish, that I should ever be another man's wife. Do you think he meant anything serious by that? PAULINE.

